# Le délai diagnostique des cancers broncho-pulmonaires vus à l'USFR de Pneumologie Befelatanana, Antananarivo, Madagascar

**DOI:** 10.11604/pamj.2019.33.263.18695

**Published:** 2019-07-29

**Authors:** Kiady Ravahatra, Michel Tiaray Harison, Oninala Fenitra Rakotondrasoa, Emeline Lucie Ramirana, Iantsotiana Davidson Rakotondrabe, Marie Odette Rasoafaranirina, Anjara Mihaja Nandimbiniaina, Jocelyn Robert Rakotomizao, Joelson Lovaniaina Rakotoson, Rondro Nirina Raharimanana

**Affiliations:** 1 Service de Pneumologie Centre Hospitalier Universitaire de Fenoarivo, Antananarivo Madagascar; 2 Service de Pneumologie Centre Hospitalier Universitaire de Befeletanana, Antananarivo Madagascar; 3 Service de Pneumologie Centre Hospitalier Universitaire de Fianarantsoa, Fianarantsoa, Madagascar

**Keywords:** Cancer, pulmonaire, Madagascar, délai diagnostique, Cancer, pulmonary, Madagascar, diagnostic delay

## Abstract

Le délai entre l'apparition des premiers signes cliniques et le diagnostic du cancer bronchique doit être le plus court possible afin que l'on puisse le prendre en charge de façon précoce et efficace. À Madagascar ce délai diagnostique reste inconnu d'où cette étude qui a pour objectif d'évalué le délai diagnostique des cancers broncho-pulmonaires vu à l'USFR de Pneumologie Befelatanana, Antananarivo, Madagascar. Il s'agit d'une étude rétrospective, descriptive portant sur les cancers broncho-pulmonaires diagnostiqués au sein de l'USFR de Pneumologie Befelatanana pendant la période du 1^er^ janvier 2011 au 31 décembre 2015 (5 ans). Nous avons inclus dans l'étude tous les patients hospitalisés pendant la période d'étude et ayant un diagnostic de cancer broncho-pulmonaire avec preuve anatomopathologique. Durant la période de notre étude, nous avons colligé 43 patients présentant un cancer broncho-pulmonaire confirmé histologiquement, soit 0,64% des patients hospitalisés. Le délai pré-hospitalier, le délai hospitalier et le délai total étaient respectivement de 171,74 jours, 13,97 jours et 185,71 jours. Le délai entre l'apparition des premiers symptômes et la consultation chez un médecin était moins de trois mois dans 30 cas (69,76%), entre 3 et 6 mois dans 8 cas (18,60%). Notre délai pré-hospitalier chez nos patients était très long par rapport à la recommandation internationale sur le délai diagnostique des cancers broncho-pulmonaire. Un effort doit être fait à ce niveau pour améliorer le délai diagnostique.

## Abstract (en)

The time between clinical symptoms onset and the diagnosis of bronchial cancer should be as short as possible so that it can be managed early and effectively. In Madagascar, this diagnostic delay is unknown. Therefore this study aims to evaluated the diagnostic delay of bronchopulmonary cancers at the the USFR Befelatanana Pneumology, Antananarivo, Madagascar. We conducted a retrospective descriptive study of patients with bronchopulmonary cancers diagnosed at the USFR Befelatanana Pneumology over the period 1^st^ January 2011 to 31^st^ December 2015 (5 years). All patients hospitalized during the study period and receiving anatomopathologically-confirmed diagnosis of bronchopulmonary cancer were included in the study. During the study period we collected data from 43 medical records of patients with bronchopulmonary cancer confirmed histologically, reflecting a rate of 0.64% of hospitalized patients. Pre-hospital delay, hospital delay and total delay were 171.74 days, 13.97 days and 185.71 days respectively. The time between symptom onset and consultation with a doctor was less than three months in 30 cases (69.76%), between 3 and 6 months in 8 cases (18.60%). Pre-hospital delay was very long according to the international recommendation for delayed diagnosis of bronchopulmonary cancers. An effort should be made to improve the diagnostic delay.

## Introduction

Le délai entre l'apparition des premiers signes cliniques et le diagnostic du cancer broncho-pulmonaire doit être le plus court possible pour une prise en charge thérapeutique précoce et efficace. Dans la majorité des cas, le diagnostic est tardif, uniquement 15 à 20% des cancers broncho-pulmonaires sont opérables au moment de leur diagnostic. Ceci est dû, en grande partie, aux signes cliniques qui sont, le plus souvent, peu spécifiques. De plus, son pronostic est mauvais avec une survie tous stades confondus de 15% à 5 ans. La survie étant meilleure pour les stades localisés que pour les stades déjà avancés [1]. Il est important de diagnostiquer et de traiter ce cancer le plus rapidement possible afin d'améliorer la survie des patients. À Madagascar, on ne connaît pas encore le délai diagnostique des cancers broncho-pulmonaires car aucune étude n'a été réalisée dans ce sens. Notre objectif est alors d'évaluer le délai diagnostique des cancers broncho-pulmonaires vus à l'USFR de Pneumologie Befelatanana.

## Méthodes

Il s'agit d'une étude rétrospective, descriptive sur les délais diagnostiques des cancers broncho-pulmonaires diagnostiqués à l'Unité de Soins, de Formation et de Recherche de Pneumologie Befelatanana, Antananarivo, Madagascar, pendant la période du 1^er^ janvier 2011 au 31 décembre 2015 (5 ans). Nous avons inclus dans l'étude tous les dossiers des patients hospitalisés pendant la période d'étude et présentant un diagnostic de cancers broncho-pulmonaires avec des preuves anatomo-pathologiques. Les paramètres suivants ont été définis pour les différents délais: délai pré-hospitalier correspondant au délai entre l'apparition des premiers signes et l'arrivé à l'hôpital, délai hospitalier correspondant au délai entre l'hospitalisation et l'obtention du diagnostic histologique, délai total correspondant à la somme des deux délais c'est-à-dire délai prés hospitalier et délai hospitalier. L'étude comprend initialement un établissement du protocole d'étude et d'une fiche de recueil des données suivie d'une consultation des registres des patients hospitalisés. Un inventaire des dossiers dont le diagnostic comprenait un cancer broncho-pulmonaire a été réalisé, suivi du remplissage de la fiche de recueil et la saisie des données sur le logiciel Excel^®^ 2013. Nous avons utilisé Microsoft Excel 2013 pour la saisie des données. L'étude statistique a été faite avec le logiciel EPI info 7.

## Résultats

Durant la période de notre étude, 6715 patients étaient hospitalisés dont 43 patients présentant un diagnostic de cancer broncho-pulmonaire confirmé histologiquement, soit 0,64% des patients hospitalisés. Le délai entre l'apparition des premiers symptômes et la consultation d'un médecin était moins de trois mois dans 30 cas (69,76%) et entre 3 et 6 mois dans 8 cas (18,60%) (Tableau 1). Les patients ont bénéficiés d'une radiographie du thorax après 1,48 jours d'hospitalisation, d'un scanner thoracique après 4,74 jours, d'une fibroscopie bronchique avec biopsie après 5,73 jours et 11,14 jours pour la ponction trans-pariétale. Le résultat de l'examen anatomopathologique des pièces de biopsies était obtenu au bout de 7,07 jours après la réalisation de la fibroscopie bronchique et après 8,42 jours pour la ponction trans-pariétale. Le délai pré-hospitalier, le délai hospitalier et le délai total était respectivement de 171,74 jours, 13,97 jours, et de 191,86 jours. La durée moyenne d'hospitalisation des patients était de 14,08 jours. Le reste des différents délais diagnostiques est présenté dans le Tableau 2. Le délai diagnostique en fonction de la symptomatologie des patients est présenté dans le Tableau 3 et le délai diagnostique en fonction du stade de cancer présenté dans le Tableau 4.

**Tableau 1:** Répartition du délai de la première consultation

Délai de première consultation	Effectifs n (%)
<3 mois	30 (69,77)
3–6 mois	8 (18,60)
>6 mois	5 (11,63)

**Tableau 2:** Répartition des différents délais diagnostiques

Type de délai	Délai moyen (jours)
Délai hospitalisation et radiographie du thorax	1,48
Délai hospitalisation et scanner thoracique	4,74
Délai hospitalisation et fibroscopie bronchique	5,73
Délai hospitalisation et ponction trans pariétale	11,14
Délai fibroscopie et résultat anatomopathologique	7,07
Délai ponction trans-pariétale et résultat anatomopathologique	8,42
Délai pré hospitalier	171,74
Délai hospitalier	13,97
Délai total	185,71
Durée moyenne d’hospitalisation	14,08

**Tableau 3:** Répartition des signes selon le délai diagnostique

signes	Effectifs n(%)	délai pré-hospitalier (jours)	délai hospitalier (jours)	délai total (jours)
dyspnée	29(67,44)	151,03	15,1	172,31
hémoptysie	9(19,56)	250	13,88	270,44
douleur thoracique	18(41,86)	182,22	14,5	202,16
Toux	35(81,39)	171,28	13,02	190,6

**Tableau 4:** Répartition des délais selon le stade des cancers

Stade du cancer	Effectifs n(%)	délai prés hospitalier (jours)	délai hospitalier (jours)	délai total (jours)
I	0	0	0	0
II	5(11,63)	300	8,4	308,4
IIIa	3 (6,98)	130	27,66	157,66
IIIb	2(4,65)	395	1	396
IV	33(76,74)	115,34	14,36	157,06

## Discussion

Dans notre étude, le délai entre l'apparition du premier symptôme et l'admission à l'hôpital (171,74 jours) qui était relativement long. Ce délai était de 46 jours dans l'étude de Virally J *et al.* [2], 76 jours dans celle de Ouazzani *et al.* [3], et 78 jours dans celle de Jabri H *et al.* [4]. Le délai d'apparition des premiers symptômes à la visite du médecin généraliste était de 14 jours dans la série de Salomaa *et al.* [5], et de 21 jours dans l'étude de Koyi *et al.* [6]. Le délai entre la première visite du médecin généraliste à la date de prise en charge spécialisée (hospitalisation) était respectivement de 24 jours et 33 jours dans les séries de Salomaa *et al.* [5] et Koyi *et al.* [6].

Dans leurs études, Mohan *et al.* [7] a démontré que le délai pré-hospitalier était largement dépendant de la sévérité des symptômes, du niveau d'éducation des patients et des facteurs socio-économiques. Cette constatation a été observée dans notre cas, la toux, symptomatologie la plus fréquente dans notre étude était souvent négligée et banalisée par les patients. En plus, les patients consultaient les tradipraticiens ou les guérisseurs traditionnels avant de voir un médecin en cas de symptôme. Ceci avait pour conséquence de rallonger le délai diagnostique. Les signes plus alarmants comme l'hémoptysie est en faible proportion dans notre étude. A côté de ces négligences constatées chez nos patients s'ajoutaient des difficultés financières. A Madagascar, il n'existe pas de prise en charge ni d'aide financière en matière de santé, c'est le malade qui paye tous les frais de consultation, les bilans paracliniques et la prise en charge thérapeutique comme les médicaments ou la chirurgie. Le facteur d'éloignement entre le lieu d'habitation des patients et le centre de santé ou le service spécialisé n'était pas négligeable. L'amélioration du pronostic de ces patients passe donc surtout sur la réduction de ce délai pré-hospitalier très long. Par contre le délai hospitalier de 13,97 jours était court dans notre étude par rapport aux résultats des autres études: trente-un jours pour l'étude KBP-2000-CHPG [8], 38 jours pour Caroline R *et al.* [9]. Vu le stade avancé de la maladie de nos patients, la recherche du diagnostic est beaucoup plus facile, raccourcissant ainsi ce délai hospitalier. Le délai total de diagnostic (185,71jours) de notre étude était relativement très long par rapport aux autres études. Virally J *et al.* ont retrouvé l62 jours [2] et 10,35 semaines pour Zivković D *et al.* [10]. Ce long délai total est surtout lié à celui du délai pré-hospitalier qui était relativement long dans notre étude. Toutefois, Gildea *et al.* [11] rapportaitnt que la plupart des patients ont connus un délai long de 5-6 mois avant d'avoir le diagnostic définitif de cancer (93,6%) et a été diagnostiqués à des stades avancés.

Des recommandations ont été élaborées par des sociétés savantes afin de définir un délai diagnostique optimal pour les cancers broncho-pulmonaire. La première était celle de BTS *(British Thoracic Society)* [12] qui recommande un maximum de deux semaines entre la réalisation d'une radiographie thoracique, demandée par un médecin généraliste suspectant un cancer bronchique et la consultation chez un spécialiste. Un délai maximal de huit semaines entre la première consultation chez un médecin spécialiste et la thoracotomie pour les cas opérables, la radiothérapie doit être débuté si elle est urgente, dans les deux jours, si elle est radicale dans les quatre semaines et si elle est palliative dans les deux semaines. La deuxième recommandation était celle des canadiennes *(Canada Medical Association Journal)* [13] qui recommande un délai de 4 semaines entre la consultation d'un médecin et le diagnostic, un délai de 2 semaines entre le diagnostic et la chirurgie. La troisième recommandation était celle de *Swedish Lung Cancer Study Group* [14] qui recommande que dans 80% des cas, toutes les investigations diagnostiques doivent être achevées dans les quatre semaines suivant la première consultation chez un spécialiste et que le traitement doit être initié dans les deux semaines qui suivent. Pour notre cas, les délais diagnostiques ont largement dépassé ces normes, 185,71 jours pour le délai global de diagnostic et 171,74 jours pour le délai pré hospitalier. Les cancers broncho-pulmonaires constituent un problème de santé publique majeur et malheureusement, la plupart est vue tardivement. Face à cette situation, nous suggérons la création d'un protocole national de dépistage des cancers broncho-pulmonaires des sujets à risques, création d'un programme de lutte contre les facteurs de risque des cancers broncho-pulmonaires (tabagisme, exposition professionnelle, pollution)

## Conclusion

Le délai diagnostique des cancers broncho-pulmonaires est un élément clé dans sa prise en charge. Il doit être le plus court possible pour espérer une prise en charge précoce et efficace. Pour notre étude, c'est au niveau du délai pré-hospitalier que nous devons agir pour diminuer ce délai diagnostique. Par contre le délai hospitalier de notre étude se trouve dans la fourchette de la recommandation internationale sur le délai diagnostique des cancers broncho-pulmonaires.

### État des connaissances actuelles sur le sujet

- Le délai diagnostique joue un rôle important dans la prise en charge et le pronostic des cancers pulmonaire;
- Le délai diagnostique doit être le plus court possible pour améliorer le pronostic des patients.

### Contribution de notre étude à la connaissance

- Cette étude permettra de connaitre le délai diagnostique des cancers pulmonaire à Madagascar;
- La connaissance de ces délais diagnostiques permet de trouver les différents points à améliorer pour réduire ce délai diagnostique.

## Conflits des intérêts

Les auteurs ne déclarent aucun conflit d'intérêts.
